# Association between serum Klotho concentration and hypertension in postmenopausal women, a cross-sectional study from NHANES 2013–2016

**DOI:** 10.1186/s12877-023-04191-8

**Published:** 2023-08-02

**Authors:** Jingli Yu, Jinfeng Li, Mingxia Li, Ling Wang, Xia Xu, Miao Li

**Affiliations:** Department of Physiological Obstetrics, Zhu Ma Dian Central Hospital, Women and Children’s Hospital, No.747 Zhonghua Road, Yicheng District, Zhu Ma Dian City, Henan Province China

**Keywords:** Klotho, Postmenopausal, Hypertension, NHANES

## Abstract

**Background:**

The objective of this study was to examine the correlation between serum Klotho protein concentration and postmenopausal hypertension.

**Methods:**

A cross-sectional study design was used, in which 1713 postmenopausal women who participated in the National Health and Nutrition Examination Survey (NHANES) 2013–2016 were included. Multivariate logistic regression models were applied to assess the association between serum Klotho concentration and postmenopausal hypertension.

**Results:**

A weighted analysis was executed, revealing a noteworthy hypertension prevalence rate of 53.44% among the study participants. Participants with lower quartile of serum Klotho concentration had a higher prevalence of hypertension than those in higher quartiles (Q1:62.29% vs. Q2: 48.52% vs. Q3: 47.33% vs. Q4: 55.02%, *p* < 0.001). Furthermore, a multivariate logistic regression analysis confirmed that participants with higher quartiles of serum Klotho concentration had a significantly reduced risk of postmenopausal hypertension compared to those in the lowest quartile. Subgroup analysis displayed consistent findings in those following subgroups: aged ≥ 65 years, obesity, nonsmokers, individuals without diabetes and coronary heart disease, and those with higher levels of estradiol and estimated glomerular filtration rate. Based on the results, we concluded that there is a significant association between serum Klotho concentration and postmenopausal hypertension.

**Conclusion:**

The findings of this study revealed a significant inverse association between serum Klotho concentration and hypertension among postmenopausal women. Serum Klotho concentration may serve as a valuable biomarker for risk stratification in postmenopausal women who are at risk of developing hypertension.

**Supplementary Information:**

The online version contains supplementary material available at 10.1186/s12877-023-04191-8.

## Introduction

Hypertension is a major contributor to morbidity and mortality around the world [1], particularly in developed countries, and place a substantial strain on healthcare and economic systems. The likelihood of developing hypertension increases significantly as both men and women age [2]. Analysis of data from the 2019 Global Burden of Disease Program revealed that the prevalence of hypertension in 2019 was nearly 2.5 times higher than in 1990 [3]. Given the rapid global aging, the burden of hypertension is expected to continually grow in the coming decades. Furthermore, persistent hypertension is a significant risk factor for a range of cardiovascular diseases, including stroke [4], heart failure (HF) [5], and arrhythmias such as atrial fibrillation (AF) [6].

Gender plays a significant role in the prevalence of hypertension, with postmenopausal women exhibiting a higher incidence of hypertension when compared to those age-matched men [7]. Globally, approximately 25% of adult women exhibit signs of hypertension [8], with an increase to 75% among individuals aged 60 years or higher in the USA [9]. Despite this relatively high hypertension prevalence in postmenopausal women, the precise underlying mechanisms remain complex and not fully comprehended. Nevertheless, research accumulation has identified several risk factors and mechanisms linked to postmenopausal hypertension, including activated sympathetic activity resulting from obesity [10], decreased estrogen level [11]and upregulated renin-angiotensin system (RAS) activity [12]. Moreover, postmenopausal women display less well-controlled blood pressure (BP) [13] and exhibit a higher tendency to experience nocturnal non-dipping BP [14] and severe target organ damage in comparison to men, highlighting the paramount importance of in-depth investigations into potential mechanisms responsible for postmenopausal hypertension.

The Klotho protein, which is mainly produced by kidneys and is considered an anti-aging protein, has been shown to correlate with a reduction in cardiovascular diseases risk in adults [15]. Furthermore, Klotho gene-overexpressing rats have shown significant lifespans extensions [16]. Prior research has confirmed that Klotho protein plays an essential role in regulating BP. A recent study has revealed a positive association between higher circulating Klotho protein levels and a decreased incidence of hypertension in older adults aged 70–79 years [17]. Nevertheless, the association between serum Klotho protein concentration and postmenopausal hypertension remains unclear. Thus, the present study aims to examine the potential linkages between circulating Klotho levels and postmenopausal hypertension, utilizing nationally representative data available from the National Health and Nutrition Examination Survey (NHANES).

## Methods

### Study design and population

This study is a cross-sectional investigation that utilizes data from the NHANES project. The NHANES project is a population-based and cross-sectional survey initiated by the National Center for Health Statistics of the Centers for Disease Control and Prevention. It is designed to assess the general health and nutritional status of the US population. The NHANES data collection process comprises a sophisticated, multi-level sampling approach that gathers crucial demographic information, physical examination details, laboratory blood and urine sample results, and health questionnaires. Our findings provide key insights into the health and nutrition status of the US population.

**Fig. 1:**
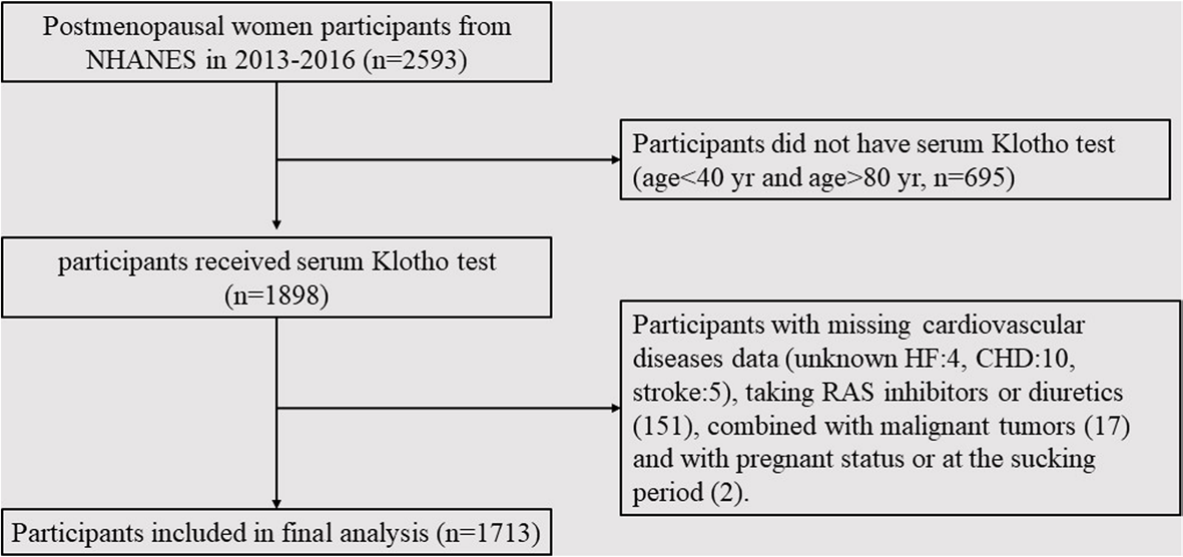
Flowchart of participants selection

This study utilized data extracted from NHANES survey cycles conducted in 2013–2014 and 2015–2016. A total of 2593 postmenopausal participants were enrolled between 2013 and 2016, excluding 695 women with missing data of serum Klotho concentration. Moreover, participants with an unknown history of cardiovascular comorbidities, including HF (4), coronary heart disease (CHD) (10), and stroke (5), were also excluded from this study (as depicted in Fig. 1). The survey protocol was approved by the NCHS Institutional Review Board, and written informed consent was obtained from all participants. Eligibility criteria were rigorous, requiring participants to be over 18 years old with postmenopausal status, and with complete data from interviews, laboratory tests, and physical examinations. Exclusion criteria were also clearly defined to exclude participants without serum Klotho concentration data, those who were taking RAS inhibitors and diuretics for BP control (151), those with combined malignant tumors (17), and those with pregnant status or at the sucking period (2).

### Measurement of serum Klotho protein concentration

As part of our analysis, participants provide fasting blood samples which were initially stored at -80 °C in the Centers for Disease Control and Prevention, Atlanta, GA for subsequent analysis. The Northwest Lipid Metabolism and Diabetes Research Laboratories, University of Washington in Seattle, WA transported the samples on dry ice for analysis. To determine serum Klotho concentration, a commercially available Elisa kit (IBL International, Japan) with a sensitivity of 6 pg/ml was utilized. Among 114 ostensibly healthy donors, the mean serum Klotho concentration value was determined to be 698.0 pg/ml, with a range of 285.8 to 1638.6 pg/ml. Duplicate analysis was conducted for each sample and the mean result was applied as the final value. Quality control measures were employed, and in cases where duplicate values were detected in over 10% of the samples, repeated examination was performed. In cases where the value of a quality control sample exceeded 2 standard deviations (SD) of the assigned value, the entire analytical process was deemed inadequate and repeated accordingly. The laboratory adhered to the manufacturer's protocol and standardized criteria through the analytic procedures.

### Definition of hypertension

BP measurements were taken from participants during the physical examination visit at the mobile examination center (MEC) through the NHANES project. After resting in a seated position for at least 5 min, three consecutive BP readings were obtained at the maximum inflation level. The average value of the three recordings was calculated for each participant in this study. The definition of hypertension followed the 2018 ESC/ESH Guidelines for the management of arterial hypertension [18]: wherein hypertension was diagnosed if participants met at least one of the following criteria: (1) physician diagnosis prior to the examination; (2) antihypertensive medication intake; (3) systolic BP ≥ 140 mmHg; (4) diastolic BP ≥ 90 mmHg.

### Evaluation of menopausal status

The menopausal status in this study was assessed using a two-step questionnaire from the reproductive health section. Firstly, participants were asked whether they had experienced a menstrual period within the previous 12 months. Subsequently, if the answer was negative, they were asked to provide a reason for the absence of menstruation during that period. Women were classified as postmenopausal if they responded affirmatively with either “menopausal” or “hysterectomy” to the latter question.

### Covariate data collection

This study collected various covariate data, which were based on the previous published literature [19, 20], clinical practice experience and the statistical significance of reason. The demographic information that was collected by standard questionnaires, including age, race/ethnicity, education level, smoking status, and the ratio of family income to poverty. Physical examination data, including weight, height, body mass index (BMI), and laboratory tests, including fasting glucose (mmol/L), high-density lipoprotein (HDL: mmol/L), total cholesterol (TC: mmol/L), blood urea nitrogen (BUN: mmol/L), triglycerides (TG: mmol/L), uric acid (umol/L), estradiol (pg/mL), testosterone (ng/dl), glycohemoglobin (HbA1c) and oral glucose tolerance test (OGTT) (two-hour glucose: mmol/L) were also collected. The estimated glomerular filtration rate (eGFR: ml/min/1.73m^2^) was calculated according to the following equation [21]: GFR = 141 × min(Scr/κ, 1)α × max(Scr/κ, 1)-1.209 × 0.993Age × 1.018 [if female] _ 1.159 [if black], where Scr is serum creatinine, κ is 0.7 for females and 0.9 for males, α is -0.329 for females and -0.411 for males, min indicates the minimum of Scr/κ or 1, and max indicates the maximum of Scr/κ or 1. Medication use including aspirin, hypoglycemic agents, statins and female hormones was obtained by a standard questionnaires. The diagnosis of cardiovascular diseases was based on self-reported answers to questions related to HF/CHD/stroke. Diabetes diagnosis was made if participants met at least one of the following criteria: (1) fasting glucose ≥ 7.0 mmol/L; (2) self-reported doctor diagnosis; (3) use of oral lowing glucose medications or insulin; (4) plasma glucose > 11.1 mmol / L for two hours of OGTT; (5) HbA1c > 6.5% [22].

### Statical analysis

Data analysis in this study adhered to the analytical guidelines prescribed by the National Center for Health Statistics. The sampling methodology employed and sample weights were considered to ensure reliable results. Participant serum Klotho levels were grouped into four quartiles (Q1 ≤ 639.70 pg/mL, Q2 639.70–782.20 pg/mL, Q3 782.20–975.50 pg/mL, and Q4 ≥ 975.50 pg/mL) for the purposes of analysis. Normally distributed continuous variables were tested with a weighted one-way ANOVA and the mean ± SD was used for presentation. Non-normally distributed continuous variables were subjected to a weighted Kruskal–Wallis tests and presented as median (interquartile range). Categorical variables were presented as weighted percentages and statistical significance was determined using weighted chi-square test. Univariate and multivariate weighted logistic regression models (Models 1 to 3) were utilized to investigate the potential link between Klotho concentration and postmenopausal hypertension. Significant variables in the univariate regression analysis with *p* < 0.05 or those with clinical significance (if *p* ≥ 0.05) were selected for use in the multivariate regression analysis. Model 1 was a crude model, and Model 2 was adjusted for age, race/ethnicity, educational level, smoking status, ratio of family income to poverty, BMI, HDL, TC, TG, BUN, e-GFR, uric acid, estradiol and testosterone. Model 3 was further adjusted for diabetes, HF, CHD, stroke and medication use (aspirin, statins, hypoglycemic agents and female hormone). Furthermore, subgroup analyses were also conducted to validate the association between Klotho and hypertension among different subgroups based on the clinical significance, multivariate logistic regression analysis and literatures reports [19, 23]. The subgroups were categorized by age, BMI, smoking status, diabetes, CHD, estradiol and renal function. Interaction tests were also carried out to assess the individualized effects of serum Klotho on hypertension across the subgroups. Restricted cubic splines were utilized to examine the non-linear association and dose–response relationship between serum Klotho concentration and postmenopausal hypertension. STATA software (version 14.0, Stata Corporation) and R statistics (version 4.0.3, R Core Team 2020) were used for all statistical analyses, and a p-value of less than 0.05 was considered statistically significant.

## Results

### Baseline characteristics of postmenopausal women participants according to the quartiles of serum Klotho protein concentration

The present study primarily investigated the association between serum Klotho concentration and hypertension in postmenopausal women. The final analysis included 1713 participants who had completed menopause and had a mean age of 61.26 ± 8.86 year. Using weighted analysis, the prevalence rate of hypertension was found to be 53.44%. The study evaluated participating individual’s baseline characteristics by the quartiles of serum Klotho concentration, which were explicated in Table 1. Compared to individuals in the higher quartiles, those in the first quartile of Klotho concentration were more likely to demonstrate an advanced age, smoking cigarettes, have Caucasian ethnicity, fewer had educational qualifications at college or beyond, higher levels of BMI, TC, TG, BUN and uric acid, and lower levels of e-GFR. Moreover, individuals in the first quartile had a higher likelihood of being affected by disorders like hypertension, diabetes, CHD, and stroke, and were also more likely to take medications than those in higher quartiles.

**Table 1:** Baseline characteristics of post-menopausal participants according to the quartiles of serum Klotho concentration

	Q 1	Q 2	Q 3	Q4	*p*-value
Age (years)	62.24 ± 8.99	61.49 ± 8.82	60.57 ± 8.75	60.57 ± 8.76	< 0.001
BMI (kg/m2)	30.58 ± 6.71	30.08 ± 7.69	29.65 ± 7.34	29.96 ± 7.96	< 0.001
Smoking status (%)					< 0.001
Current	18.28	16.04	17.99	13.47	
former	31.56	29.37	28.11	25.93	
never	50.17	54.59	53.90	60.60	
Race/ ethnicity (%)					< 0.001
Mexican American	4.74	4.73	7.58	5.83	
Other Hispanic	4.38	3.57	4.61	5.33	
Non-Hispanic White	77.54	78.77	73.39	70.30	
Non-Hispanic Black	7.25	6.69	7.50	11.59	
Others, including multi-racial	6.09	6.24	6.92	6.95	
Education (%)					< 0.001
Less than high school	13.59	8.77	13.92	13.27	
High school or equivalent	26.57	23.02	21.18	16.22	
College or above	59.85	68.21	64.90	70.51	
Family poverty income ratio	3.05 ± 1.60	3.14 ± 1.63	3.08 ± 1.60	3.08 ± 1.71	< 0.001
Medication use					
Antithrombotic agents (%)	30.62	31.75	27.02	24.51	< 0.001
Hypoglycemic agents (%)	17.75	12.80	14.77	13.08	< 0.001
Statins (%)	32.20	34.77	27.00	30.04	< 0.001
HRT (%)	49.08	41.42	39.77	42.13	< 0.001
SBP	126.19 ± 17.05	126.82 ± 15.65	123.80 ± 19.26	126.27 ± 17.11	< 0.001
DBP	68.88 ± 10.50	68.05 ± 12.00	70.06 ± 12.14	68.99 ± 11.28	< 0.001
Hypertension (%)	62.29	48.52	47.33	55.02	< 0.001
Diabetes (%)	25.68	20.28	24.61	22.98	< 0.001
HF (%)	2.66	2.41	5.18	3.57	< 0.001
CHD (%)	4.63	4.48	3.70	4.17	< 0.001
Stroke (%)	5.16	5.29	5.07	2.08	< 0.001
HDL (mmol/L)	1.63 ± 0.62	1.61 ± 0.53	1.58 ± 0.45	1.64 ± 0.48	< 0.001
TC (mmol/L)	5.48 ± 1.08	5.53 ± 1.26	5.40 ± 0.99	5.30 ± 1.01	< 0.001
TG (mmol/L)	1.82 ± 0.99	1.84 ± 1.24	1.75 ± 1.05	1.51 ± 0.89	< 0.001
e-GFR (ml/min/1.73m2)	90.27 ± 31.76	92.81 ± 33.10	96.47 ± 36.99	101.40 ± 37.58	< 0.001
BUN (mmol/L)	5.52 ± 2.20	5.50 ± 2.31	5.27 ± 2.02	5.17 ± 1.65	< 0.001
Uric acid (umol/L)	314.15 ± 82.32	301.95 ± 81.85	294.39 ± 73.58	276.74 ± 69.00	< 0.001
Estradiol (pg/ml)	6.30(3.36, 11.70)	6.28 (3.23, 10.90)	6.10 (3.20, 10.30)	6.35 (3.77, 11.60)	< 0.001
Testosterone (ng/dl)	16.10(10.10, 24.70)	16.80 (11.00, 23.60)	15.41 (10.60, 24.05)	17.10 (11.40, 23.60)	< 0.001

*BMI* Body mass index, *HRT* Hormone replacement therapy, *SBP* Systolic blood pressure, *DBP* Diastolic blood pressure, *HF* Heart failure, *CHD* Coronary heart disease, *HDL* High-density lipoprotein, *TC* Total cholesterol, *TG* Triglyceride, *eGFR* Estimated glomerular filtration rate, *BUN* Blood urea nitrogen

### Association between quartiles of serum Klotho concentration and hypertension in postmenopausal participants

**Fig. 2:**
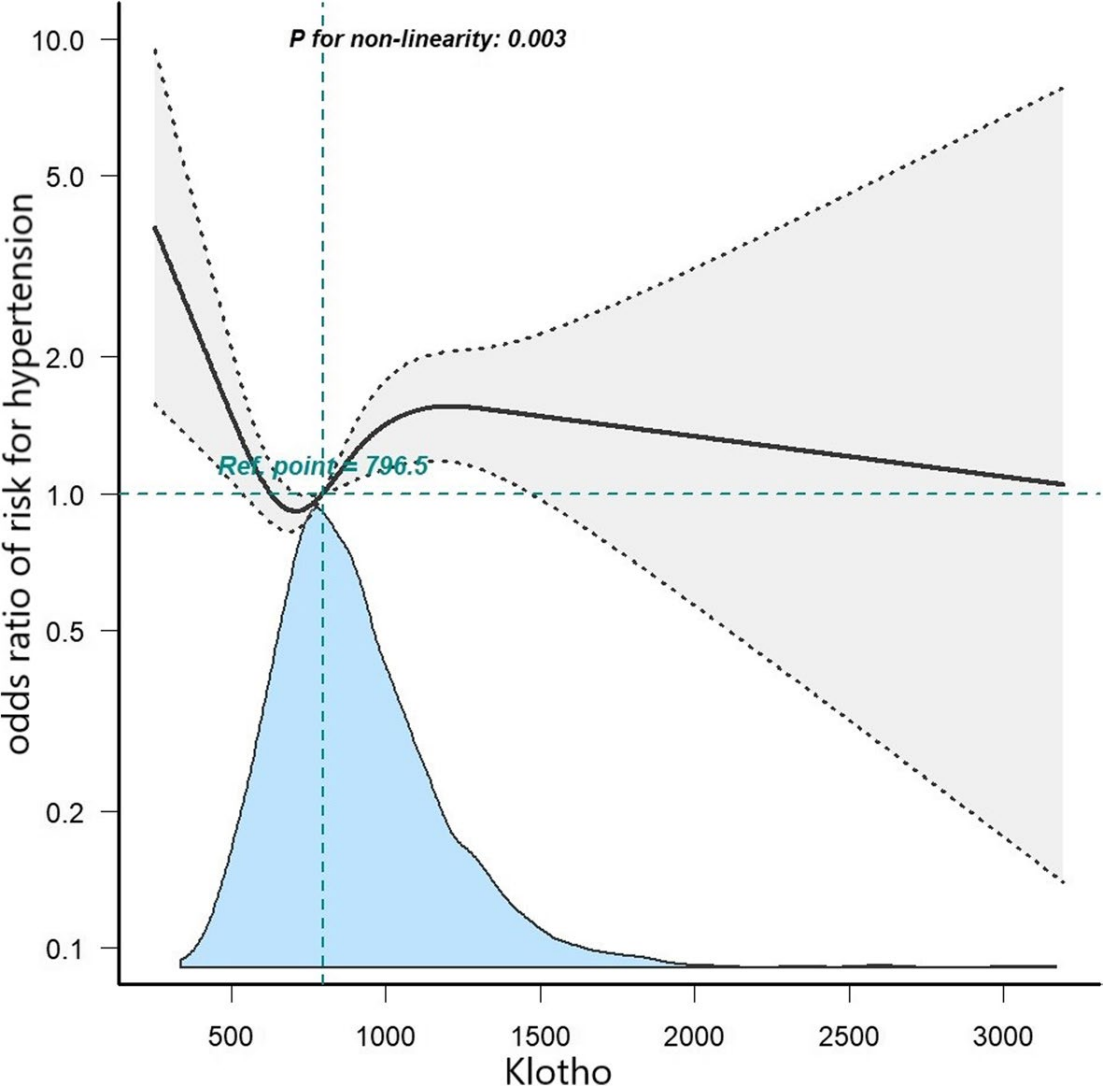
The non-linear association and dose–response relationship between serum Klotho concentration and hypertension assessed by restricted cubic spline regression. Model was adjusted for age, race/ethnicity, educational level, smoking status, BMI, family poverty income ratio, HDL, TC, TG, BUN, e-GFR, uric acid, estradiol, testosterone, diabetes, HF, CHD, stroke and medication use

To examine the possible association between serum Klotho protein concentration and hypertension in postmenopausal women, weighted logistic regression models were performed. The results of the regression analysis regarding the association between quartiles of serum Klotho concentration and postmenopausal hypertension are presented in Table 2. After fully adjusting for potential confounders, a significant inverse relationship between serum Klotho concentration and hypertension was observed. Postmenopausal women with serum Klotho protein concentration in the second and third quartiles had a reduced probability of 50% and 39% of being susceptible to hypertension compared to those in the lowest quartile. Furthermore, a restricted cubic spline analysis confirmed a significant nonlinear association between serum Klotho concentration and postmenopausal hypertension. The dose–response association demonstrated a trend of decreasing initially, followed by an increase and then another decrease in hypertension risk with an increase in Klotho concentration, characterized by a reflection point for serum Klotho concentration at 796.5 pg/ml (Fig. 2, *p* for non-linearity 0.003).

**Table 2:** Association between quartile of serum Klotho concentration and hypertension among American postmenopausal women participants

	Model1	Model2	Model3
Klotho (quartiles)			
Q1	Reference	Reference	Reference
Q2	0.57 (0.39–0.83) 0.004	0.60 (0.38–0.95) 0.029	0.50 (0.30–0.82) 0.006
Q3	0.54 (0.37–0.79) 0.002	0.60 (0.39–0.93) 0.021	0.61 (0.38–0.97) 0.038
Q4	0.74 (0.51–1.09) 0.124	0.88 (0.57–1.36) 0.558	0.89 (0.56–1.40) 0.603
*p* value	0.005	< 0.001	< 0.001

Data were present as odds ratio (*OR*) and 95% confidence interval (*CI*). Model1 was adjusted for none. Model2 was adjusted for age, body mass index (*BMI*), smoking status, family poverty income ratio, race/ethnicity, education level, blood urea nitrogen, uric acid, high density lipoprotein, total cholesterol, triglycerides, estimated glomerular filtration rate, estradiol and testosterone. Model3 was further adjusted for diabetes, coronary heart disease, heart failure, stroke and medication use (including aspirin, statins, hypoglycemic agents and female hormone)

### Subgroup analysis

We also conducted a subgroup analysis to further investigate the reciprocal relationship between serum Klotho protein concentration and hypertension for postmenopausal women. The findings in Table 3 highlight that in the following subgroups: subjects aged over 65 years, obesity, nonsmokers, individuals without diabetes and CHD, and those with higher levels of estradiol (≥ 6.22 pg/mL) and e-GFR (≥ 60 mL/min/1.73m2), the risk of hypertension were significantly lower in the higher quartiles of serum Klotho protein concentration when compared to participants in the first quartile.

**Table 3:** Subgroup analysis of the association between serum Klotho concentration and hypertension in post-menopausal participants

Subgroup	Q1	Q2	Q3	Q4	*p* for interaction
		OR (95%CI)	OR (95%CI)	OR (95%CI)	
Age					0.012
< 65 years	Reference	0.58 (0.31–1.11)	0.61 (0.33–1.14)	0.66 (0.35–1.21)	
≥ 65 years	Reference	0.32 (0.15–0.69)*	0.61 (0.329–1.28)	1.38 (0.66–2.87)	
BMI					< 0.001
≥ 30 kg/m^2^	Reference	0.32 (0.16–0.62) *	0.42 (0.22–0.82) *	0.87 (0.41–1.85)	
< 30 kg/m^2^	Reference	0.65 (0.29–1.43)	0.79 (0.38–1.62)	1.01 (0.49–2.06)	
Smoking					0.001
Never	Reference	0.36 (0.18–0.74) *	0.50 (0.25–0.99) *	0.56 (0.30–1.03)	
Former	Reference	0.81 (0.28–2.31)	0.69 (0.26–1.83)	2.27 (0.94–5.50)	
Current	Reference	0.34 (0.10–1.10)	0.70 (0.20–2.44)	0.85 (0.22–3.30)	
Diabetes					< 0.001
Yes	Reference	0.58 (0.22–1.50)	0.41 (0.15–1.13)	0.88 (0.32–2.41)	
No	Reference	0.48 (0.26–0.86) *	0.62 (0.36–1.07)	0.90 (0.54–1.54)	
CHD					< 0.001
Yes	Reference	0.13 (0.01–2.20)	0.56 (0.02–14.17)	0.03 (0.00–2.13)	
No	Reference	0.50 (0.30–0.83) *	0.60 (0.37–0.97) *	0.95 (0.60–1.49)	
Estradiol concentration					0.288
< 6.22 pg/ml	Reference	0.56 (0.28–1.13)	0.84 (0.43–1.64)	1.42 (0.75–2.70)	
≥ 6.22 pg/ml	Reference	0.46 (0.23–0.91) *	0.42 (0.22–0.83) *	0.63 (0.33–1.20)	
Renal function					< 0.001
eGFR < 60 mL/min/1.73m^2^	Reference	1.61 (0.51–5.07)	0.46 (0.12–1.74)	1.08 (0.32–3.67)	
eGFR ≥ 60 mL/min/1.73m^2^	Reference	0.41 (0.24–0.68) *	0.63 (0.38–1.04)	0.89 (0.54–1.46)	

The analysis was adjusted for age, body mass index (*BMI*), smoking status, race/ethnicity, education level, family poverty income ratio, high-density lipoprotein (*HDL*), total cholesterol (*TC*), Triglyceride (*TG*); estimated glomerular filtration rate (*eGFR*), blood urea nitrogen (*BUN*), uric acid, estradiol, testosterone, diabetes, coronary heart disease (*CHD*), stroke, heart failure (*HF*) and medication use (aspirin, statins, hypoglycemic agents and female hormone)

^*^p < 0.05

## Discussion

This study represents the inaugural examination of the association between serum Klotho protein concentration and hypertension within the postmenopausal women of the US population. Our primary outcomes measures revealed two key results, as follows: (1) we identified an inherent association between serum Klotho concentrations and postmenopausal hypertension, independent of traditional risk factors; (2) we demonstrate a significant non-linear relationship between hypertension and serum Klotho concentration.

### Prevalence of hypertension

Hypertension, a prominent cause of mortality, and is closely related to the development of various cardiovascular disorders [24] and chronic kidney disease [25]. The prevalence rates of hypertension differ among men and women [12]. For men, hypertension prevalence generally increases with advancing age. However, for women, the perimenopausal period is a crucial time for the onset of hypertension. Based on previous studies, premenopausal women experience significantly lower hypertension rates than age-matched men, while it was similar or higher in postmenopausal women. To evaluate the association between serum vitamin D concentration and hypertension in pre- and postmenopausal women, a recent study was conducted in the American women. The study revealed a four-fold higher hypertension prevalence rate in postmenopausal women than premenopausal women (58.2% vs 14.7%, *p* < 0.001) [26]. Thus, our findings are consistent with the results of this study (53.44%).

### Vasoconstrictors

The RAS is known to play a crucial role in the development of hypertension, particularly in the aging population. Angiotensin-converting enzyme inhibitor and angiotensin II (Ang II) receptor blocker are frequently used as one of the most important classes of antihypertensive medications to control BP in clinical practice. Population-based studies have reported that renin activity of postmenopausal women is considerably up-regulated [27], and some gene polymorphisms of renin significantly associated with hypertension incidence in women aged 40–70 years, but not in men [28]. Yanes et al. investigated the effect of losartan (an angiotensin type I receptor antagonist) in spontaneously hypertensive postmenopausal rats (SHRs) and found that the medication reduced BP levels (not normalized), indicating that although not solely, the activated RAS may significantly contribute hypertension development [29]. Previous investigations have also demonstrated a significant influence of RAS activity on Klotho concentration [30]. ZLL [31] et al. observed a substantial increase in RAS components and BP, accompanied by a decrease in renal Klotho expression in a remnant kidney mouse model. However, the introduction of exogenous Klotho through hydrodynamic-based gene delivery counteracted the induction of multiple RAS proteins, including angiotensinogen, renin, angiotensin-converting enzyme, and angiotensin II type 1 receptor. Moreover, it normalized BP levels.

In addition to the regulation of the RAS, upregulated endothelin synthesis is known to contribute to hypertension development through vasoconstriction in small arteries and increased sodium absorption in the kidney. Specifically, prolonged stimulation of Ang II results in the significant upregulation of endothelin synthesis [32]. The plasma level of endothelin was found to increase in postmenopausal women, suggesting it’s potential involvement in hypertension development in this population [33]. Furthermore, recent compelling evidence supports the notion of Klotho negatively regulating endothelin expression. In patients with type-2 diabetes, Jian-Jun Liu et al. [34]. reported an inverse association between serum Klotho concentrations and endothelin-1 (ET-1) (Rho = -0.410, *p* < 0.001). Yuhong Wang et al. [35]. found a significant decrease in Klotho concentration in the kidneys of spontaneously hypertensive rats (SHRs) compared to Wistar–Kyoto (WKY) rats. Notably, Klotho gene delivery reversed the upregulation of ET-1 levels and the downregulation of endothelin type B (ETB) receptors in SHR kidneys. Endothelin function through ETA and ETB receptors, with ETA receptor primarily responsible for vasoconstriction. Inhibiting the ETA receptor led to a significant reduction in BP in postmenopausal female SHRs, but not in male rats or premenopausal younger female rats [36]. Conversely, ETB receptor mediates vasodilation via the coupling with nitric oxide (NO) in endothelial cells, and their activity was significantly linked to BP, which changes with Ang II treatment. Research by Megan M. Wenner's [37] and colleagues has shown that the vasodilation function of ETB receptors was considerably altered in postmenopausal women, with a positive association between ETB expression and brachial artery flow-mediated dilation. However, it remains unknown whether changes occur in the ETA receptor in postmenopausal women, as far as current research concerned.

Although experimental studies have widely recognized the protective effect of estrogen on the female cardiovascular system, the controversy surrounding the effect of hormone replacement therapy (HRT) in preventing postmenopausal hypertension persist. Xue [38] et al. conducted a study that showed a considerable reduction in Ang II-induced hypertension by upregulated estrogen activation in rat models. Similarly, estrogen therapy in postmenopausal women was shown to raise plasma NO levels and reduce ET-1, as reported by Patricia JM et al. [39]. Ichikawa [40] reported a decrease in diastolic and mean BP among normotensive postmenopausal women who took HRT for 1 and 2 years. However, Prelevic [41] reported no significant impact of HRT on BP or even an increased BP effect in postmenopausal women who received HRT for at least 5 years. In the present study, the weighted prevalence of HRT was slightly higher in participants with hypertension than those without (44.97% vs. 41.27%, *p* < 0.001), but no significant association was observed between HRT and hypertension risk in multivariate logistic regression analysis (Table S1). Additionally, although the estradiol was not independently associated with the postmenopausal hypertension (Table S1), we found that participants in the higher estradiol group with a higher level of Klotho concentration had a significant reduced risk of hypertension, while not in the lower estradiol group, also indicating the protective value of estrogen against hypertension.

### Obesity

Obesity is a prevalent condition in postmenopausal women, particularly in developed countries, and is strongly associated with the development of hypertension. Notably, obese individuals exhibited heightened sympathetic activity, particularly in the kidney, which stimulates RAS activation and subsequently leads to hypertension [42]. Furthermore, obesity is a key component of metabolic syndrome, which significantly affects BP. The presence of metabolic syndrome significantly attenuates the antihypertensive effect by impeding endothelial dysfunction and fostering inflammation [43]. Recent studies have also demonstrated that obesity is associated with reduced expression of Klotho. A cross-sectional study revealed that individuals with higher visceral adiposity index (VAI) score exhibited significantly lower serum Klotho concentrations [44]. Fibroblast growth factor 21 (FGF21), a member of the FGF family primarily produced by the liver, plays a well-established role in regulating metabolic status [45]. Klotho serves as an obligatory co-receptor for FGF-21. Yi-Ying Kuo et al. [46], discovered that a high-fat diet led to a significant decrease in Klotho expression in male mice, accompanied by weight gain. Treatment with FGF-21 resulted in weight loss and restored the decreased expression of Klotho induced by the high-fat diet, indicating the crucial role of FGF-21 in regulating Klotho expression during obesity development. In this study, we found that hypertensive participants had significantly higher BMI levels than their normotensive counterparts (31.80 ± 7.94 vs. 28.13 ± 6.25, *p* < 0.001). And multivariate logistic regression analysis also showed that BMI was independently associated with postmenopausal hypertension risk (OR: 1.08, 95%CI: 1.04–1.12) (Table S1). Additionally, we identified lower serum Klotho concentrations in obese participants (BMI ≥ 30 kg/m2) compared to non-obese participants (BMI < 30 kg/m2) (774.15 vs. 814.80, *p* = 0.027).

### Klotho protein

The Klotho protein has been recognized as a regulator of hypertension development through various pathways. This study aimed to examine the potential association between serum Klotho concentration and hypertension among postmenopausal women in the US. general population, and our findings demonstrate, for the first time, that participants with higher serum Klotho quartiles showed a significantly lower risk of hypertension than those in the first quartile. In another study involving well-functioning older adults, David A. Drew found that higher Klotho levels were associated with a 20% decreased risk of incident hypertension [17]. Additionally, an earlier animal study reported that Klotho protein was significantly suppressed in SHR, and introducing Klotho gene expression inhibited the progression of hypertension in SHR and alleviated renal damage [47]. Salt sensitivity is a crucial risk factor for hypertension development among aging individuals. Wakako Kawarazaki et al. suggested that Klotho deficiency leads to age-related salt-sensitive hypertension through activating the vascular non-canonical Wnt5a/RhoA pathways [48]. Furthermore, the animal study demonstrated that oxidative stress levels were elevated among postmenopausal rats, and long-term vitamin E and C antioxidative treatment reduced BP, indicating the notable contribution of oxidative stress to the development of postmenopausal hypertension [49]. Earlier research has indicated that Klotho may play a role in preventing tissue damage, including cardiac myocytes, by exerting an antioxidant effect [50]. Additionally, Klotho has an essential regulatory function in estrogen levels among women. According to a study by Toyama R et al. [51], Klotho-deficient mice exhibited impaired gonadotropin regulation, leading to atrophy of female reproductive systems and reduced estrogen synthesis. Furthermore, maintain a normal phosphate balance was shown to significantly prevented Klotho deficiency-induced HF in a recent study [52]. These findings suggest a critical interplay between soluble Klotho protein and estrogen in preventing hypertension development in females. Further research related to potential underlying mechanisms is warranted.

This study used a nationally representative sample of postmenopausal Americans with hypertension to conduct a secondary analysis, which strengthened the validity and generalizability of the findings. Demographic, examinational, and laboratory test variables were adjusted to ensure the reliability of the study results. Subgroup analysis was also conducted, which further established the robustness of the outcomes. Nonetheless, it is important to acknowledge the limitations of the study. Although a significant association between serum Klotho concentration and postmenopausal hypertension was observed, a causal relationship could not be established due to the study’s observational nature. While several confounding variables were adjusted, some potential residual factors that may affect the study outcomes were not accounted for.

## Conclusion

In summary, the serum Klotho concentration shows promise as an important predictor for risk stratification in postmenopausal women with an increased susceptibility to hypertension. Nevertheless, the precise causal relationship between serum Klotho concentration and hypertension has yet to be confirmed, and thus, it is essential to conduct further prospective cohort studies and clinical trials to elucidate this relationship.

## Supplementary Information

**Additional file 1. **

## Data Availability

The data sets used and analyzed in this study are available from the corresponding author for reasonable use. The NHANES public database is available at https://www.cdc.gov/nchs/nhanes/.

## Abbreviations

BP: Blood pressure
OGTT: Oral glucose tolerance test
BMI: Body mass index
HDL: High density lipoprotein
TC: Total cholesterol
TG: Triglyceride
eGFR: Estimated glomerular filtration rate
BUN: Blood urea nitrogen
HF: Heart failure
CHD: Coronary heart disease
HRT: Hormone replacement therapy
NHANES: National Health and Nutrition Examination Survey
RAS: Renin-angiotensin system
Ang II: Angiotensin II
SHRs: Spontaneously hypertensive postmenopausal rats
VAI: Visceral adiposity index
FGF-21: Fibroblast growth factor 21
