# Supplementary material for: Association between serum Klotho concentration and hypertension in postmenopausal women, a cross-sectional study from NHANES 2013–2016
Source: BMC Geriatr. 2023 Aug 2;23:466. doi: 10.1186/s12877-023-04191-8 (PMC10394796; doi:10.1186/s12877-023-04191-8)
Supplement: Supplementary file 1 — Additional file 1. [file 12877_2023_4191_MOESM1_ESM.docx]

table S1: Univariate and multivariate logistics regression analysis for the risk of postmenopausal hypertension

| Variables | Univariate | | Multivariate | |
| --- | --- | --- | --- | --- |
|  | OR (95%CI) | p-value | OR | p-value |
| age | 1.07 (1.05-1.08) | <0.001 | 1.05 (1.02-1.07) | <0.001 |
| BMI | 1.08 (1.06-1.10) | <0.001 | 1.08 (1.04-1.12) | <0.001 |
| Race |  | <0.001 |  |  |
| Mexican American | Reference | Reference | Reference | Reference |
| Other Hispanic | 0.91 (0.62-1.33) | 0.633 | 0.92 (0.52-1.63) | 0.770 |
| Non-Hispanic White | 0.97 (0.71-1.33) | 0.859 | 1.32 (0.81-2.17) | 0.269 |
| Non-Hispanic Black | 2.92 (1.99-4.27) | <0.001 | 3.53 (2.00-6.23) | <0.001 |
| Others, including multi-racial | 2.01 (1.28-3.17) | 0.002 | 3.35 (1.75-6.42) | <0.001 |
| Smoking status |  | 0.058 |  |  |
| Current | Reference | Reference | Reference | Reference |
| former | 0.74 (0.49-1.11) | 0.147 | 0.65 (0.36-1.16) | 0.142 |
| never | 0.64 (0.44-0.93) | 0.018 | 0.59 (0.34-0.99) | 0.047 |
| Education |  | 0.001 |  |  |
| Less than high school | Reference | Reference | Reference | Reference |
| High school or equivalent | 0.74 (0.50-1.09) | 0.130 | 0.75 (0.44-1.26) | 0.275 |
| College or above | 0.55 (0.40-0.76) | <0.001 | 0.77 (0.48-1.23) | 0.270 |
| Family poverty income ratio | 0.78 (0.72-0.85) | <0.001 | 0.92 (0.82-1.04) | 0.180 |
| Medication use |  |  |  |  |
| Antithrombotic agents | 4.38 (3.18-6.01) | <0.001 | 2.82 (1.86-4.27) | <0.001 |
| Hypoglycemic agents | 3.93 (2.61-5.94) | <0.001 | 0.98 (0.47-2.06) | 0.962 |
| Statins | 4.22 (3.06-5.82) | <0.001 | 2.30 (1.53-3.48) | <0.001 |
| HRT | 1.16 (0.89-1.53) | 0.276 | 1.10 (0.77-1.57) | 0.616 |
| Diabetes | 2.70 (1.97-3.71) | <0.001 | 1.22 (0.69-2.18) | 0.491 |
| HF | 6.67 (2.21-20.10) | 0.001 | 3.17 (0.70-14.38) | 0.136 |
| CHD | 4.67 (2.24-9.71) | <0.001 | 0.95 (0.30-2.99) | 0.928 |
| Stroke | 2.62 (1.39-4.93) | 0.003 | 0.74 (0.30-1.83) | 0.513 |
| HDL | 0.53 (0.36-0.78) | 0.001 | 0.96 (0.60-1.55) | 0.878 |
| TC | 0.80 (0.70-0.90) | <0.001 | 0.97 (0.81-1.17) | 0.773 |
| TG | 1.35 (1.18-1.54) | <0.001 | 1.24 (0.98-1.57) | 0.076 |
| e-GFR | 1.00 (0.99-1.00) | 0.295 | 1.00 (0.99-1.01) | 0.627 |
| BUN | 1.12 (1.05-1.20) | <0.001 | 1.03 (0.93-1.14) | 0.554 |
| Uric acid | 1.01 (1.00-1.01) | <0.001 | 1.00 (1.00-1.00) | 0.287 |
| Estradiol | 1.00 (0.99-1.00) | 0.867 | 1.00 (1.00-1.01) | 0.097 |
| Testosterone | 1.00 (0.99-1.00) | 0.378 | 1.00 (0.99-1.01) | 0.644 |

BMI: body mass index; HRT: hormone replacement therapy; SBP: systolic blood pressure; DBP: diastolic blood pressure; HF: heart failure; CHD: coronary heart disease; HDL: high-density lipoprotein; TC: total cholesterol; TG: Triglyceride; eGFR: estimated glomerular filtration rate; BUN: blood urea nitrogen;
